# Leaching of Di(2-ethylhexyl) Phthalate from Polyvinyl Chloride Microplastics in Synthetic Freshwater, Seawater, and Fish In Vitro Digestion Fluids

**DOI:** 10.1021/acsestwater.6c00513

**Published:** 2026-07-21

**Authors:** Rui Cai, Parul Sharma, Victoria Rhodes, Dhanuka Thennakoon, Justin Scott, Jimmie Weaver, Matteo Minghetti, Jorge Gonzalez-Estrella

**Affiliations:** † School of Civil and Environmental Engineering, 7618 Oklahoma State University, Stillwater, Oklahoma 74078, United States of America; ‡ Department of Chemistry, Oklahoma State University, Stillwater, Oklahoma 74078, United States of America; § Department of Biology, Oklahoma State University, Stillwater, Oklahoma 74078, United States of America

**Keywords:** polyvinyl chloride, polymer additives, plasticizer leaching, in vitro digestion, UV-aging, bioaccessibility

## Abstract

We investigated the leaching of additives from microplastics (MPs) derived from commercial polyvinyl chloride (PVC) wire insulation into synthetic freshwater, synthetic seawater, and a fish in vitro digestion system. The experiments used an MP loading of 1 mg mL^–1^ to meet the instrumental LOQ; therefore, the system should be interpreted as a controlled mechanistic design rather than a direct simulation of environmental MP exposure. We identified aging as a critical factor for DEHP leaching. While nonaged MPs showed no quantifiable leaching, UV-aged MPs leached DEHP instantaneously across all media. In controlled aqueous systems, elevated salinity suppressed apparent DEHP concentrations, yielding lower values in seawater (46.8 ± 3.6 μg L^–1^) compared to freshwater (63.1 ± 4.4 μg L^–1^). During in vitro digestion, ground fish food acted as a dietary sorptive matrix and reduced apparent fluid-phase DEHP concentrations. DEHP reached 50.0 ± 3.4 μg L^–1^ in the gastric phase and 49.8 ± 2.4 μg L^–1^ in the intestinal phase without added food, whereas fluids containing ground fish food showed lower concentrations of 27.9 ± 2.3 μg L^–1^ and 27.3 ± 2.0 μg L^–1^, respectively. These findings demonstrate that UV aging, salinity, and dietary matrices regulate DEHP leaching and bioaccessibility from PVC MPs.

## Graphical abstract

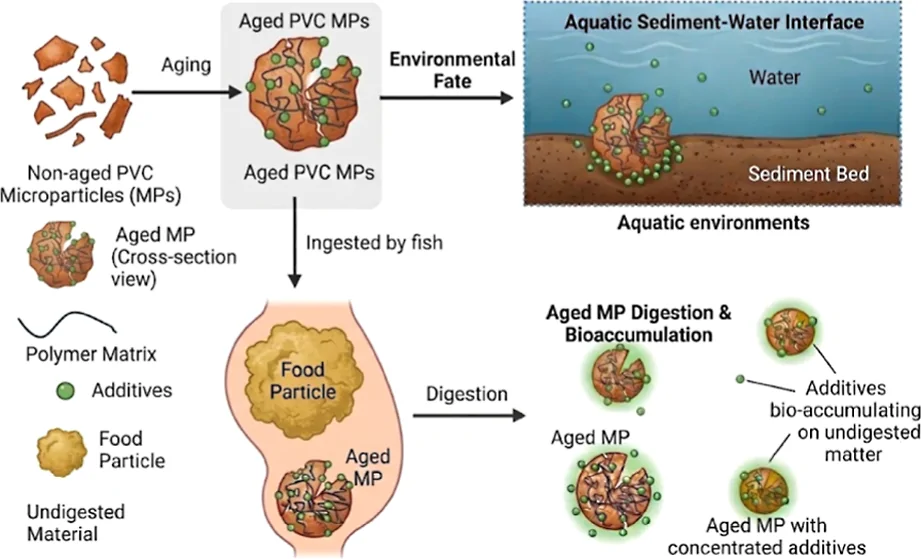

## 1 Introduction

Polyvinyl chloride (PVC) ranks among the most widely produced plastics, serving construction, automotive, packaging, electrical, and healthcare industries because of its water resistance, flame retardancy, and relatively low cost compared to other thermoplastics.^1,2^ However, the polarized carbon-chlorine bonds in PVC’s repeating units generate strong dipole–dipole interactions, making unplasticized PVC rigid and brittle. To address this limitation, manufacturers incorporate plasticizers to enhance PVC’s flexibility.^3^ The type and amount of plasticizer reflect a balance between performance requirements, cost, and regulatory considerations.^3,4^ In specific applications, such as leather simulants, the plasticizer content can reach up to ∼54.5% by weight (w/w).^5^ These plasticizers typically contain polar ester groups which form electrostatic interactions with PVC hydrogen atoms, along with bulky nonpolar segments that disrupt dipole–dipole interactions between polymer chains. Together, these effects increase the free volume within the polymer matrix, weaken the interactions between PVC molecules, and ultimately improve flexibility.^6,7^

Since external plasticizers are not chemically bound to PVC molecules, they have high mobility within the polymer matrix and can transfer into the surrounding environment,^8,9^ including leaching from PVC waste into soil and water in landfills, transfer from PVC food wraps to food, and even exposure to children through chewing on toys and school supplies.^5,10–12^ These external plasticizers can act as endocrine-disrupting chemicals. Both animal and epidemiological studies have linked them to a range of adverse health effects, including reproductive, neurological, developmental, and immune system disruptions.^13,14^

Recycling and incineration of PVC waste pose more challenges than processing other polymers due to the release of hydrogen chloride during conventional thermal degradation.^15,16^ PVC microplastics (MPs) primarily originate from improperly managed PVC waste, which undergoes natural aging processes and breaks down into particles smaller than 5 mm.^17^ PVC MPs can then enter aquatic environments via improper waste management,^18^ wastewater discharge, aerial deposition,^19^ or compromised PVC pipes and distribution systems.^20,21^ In aquatic environments, with a density of approximately 1.4 g cm^–3^, PVC MPs are likely to settle in sediment beds but can become resuspended during high flow conditions.^22^

Additionally, PVC MPs become smaller and more hydrophilic as they age. Due to the formation of oxygen-containing functional groups, which in turn, enhances plasticizer leaching into aquatic environments.^23,24^ Consequently, PVC MPs can serve as vectors, transporting plasticizers into aquatic systems. Studies have observed commonly used plasticizers, including dibutyl phthalate (DBP), di­(2-ethylhexyl) phthalate (DEHP), di­(2-ethylhexyl) terephthalate (DOTP), and diisononyl phthalate (DINP), being leached into aquatic environments via this pathway.^23,25,26^ However, the leaching of these plasticizers into aquatic environments is still not fully understood.

The small size of MPs also leads to their accidental ingestion by aquatic organisms,^27–31^ including fish. However, the correlation between ingested MPs and plasticizer levels in the organism tissues is complex, as environmental factors such as salinity and pH can influence the sorption and desorption rates of these chemicals from MPs into surrounding media.^32^ In addition, ingested MPs may undergo degradation within the digestive system.^33^ The digestive system typically consists of a gastric phase and an intestinal phase. During the gastric phase, pepsin is responsible for protein digestion in an acidic environment (pH ≈ 2). After acidic digestion, the chyme enters the intestine, where it mixes with bile, intestinal enzymes, and pancreatic secretions, further digesting proteins, carbohydrates, and lipids under mildly alkaline conditions (pH ≈ 8).^34^ Researchers have previously used in vitro digestive models to investigate the fate of ingested MPs and tire wear particles into the digestive system and the leaching of plasticizers under physiological conditions.^35–37^

In this study, we investigated the leaching of plasticizers from commercial PVC wire insulation in both aquatic environments (i.e., synthetic seawater and fresh water) and an fish in vitro digestion system.^34^ The specific objectives are (1) to identify the plasticizers present in PVC samples using gas chromatography–mass spectrometry (GC–MS) coupled with NIST library, and (2) to examine the leaching of selected plasticizers from PVC MPs in freshwater, seawater, and a fish in vitro digestion system. By using MPs generated from commercial materials, we aim to provide a more realistic representation of environmental exposure. Furthermore, by examining leaching in both aquatic and gastrointestinal media, this study offers a comprehensive assessment of plasticizer leaching associated with MP fate.

## 2 Materials and Methods

### 2.1 PVC MPs and Characterization

PVC was obtained from insulated wire purchased from a local building supply store. The PVC insulation layer was peeled off and cut into 3 mm pieces. The cut pieces were then placed into a stainless-steel grinding vial (SPEX SamplePrep 8007) for milling. The vial was first cooled in liquid nitrogen for 5 min and then placed in a ball mill (SPEX SamplePrep 8000M) for 5 min of milling. This cooling and milling cycle were repeated 10 times to ensure thorough fragmentation. After milling, the resulting PVC MPs were sieved through a 40-mesh (425 μm) stainless steel filter to obtain PVC MPs. The PVC MPs were subsequently divided into two groups for the experiment: (1) a UV-aged group and (2) a nonaged control group. For the accelerated UV-aging treatment, the PVC MPs were irradiated with 254 nm UV light in a UV chamber (Mystaire MY-DB42C) for 40 d.^38^

The surface morphology and elemental composition of the PVC MPs were characterized using a scanning electron microscope (SEM; FEI Quanta 600) equipped with an energy-dispersive X-ray spectroscopy (EDS) system (Bruker QUANTAX XFlash 6). Particle size was determined by Feret diameters measurements of more than 300 particles from SEM images using ImageJ. Changes in the functional groups of the MPs were analyzed by attenuated total reflectance Fourier transform infrared spectroscopy (ATR-FTIR; Nicolet iN10). The surface charge of the MPs was measured by ζ-potential analysis using a dynamic light scattering system (DLS; Malvern Zetasizer Advance Ultra). Prior to measurement, the MPs were suspended in pure water (pH 7.0) at 25 °C.

### 2.2 Identification of Plasticizers in MPs

A mass of 20 mg of nonaged PVC MPs and UV-aged PVC MPs was added separately to 20 mL of hexane in borosilicate glass vials, sealed with aluminum foil-lined caps to minimize contamination from plastic containers. The vial was kept at room temperature for 48 h, then filtered the solution through 1 μm glass fiber filters (Sterlitech AE2500) to remove remaining PVC MPs. The filtered extract was analyzed using a GC–MS (Agilent 8890 GC/5975 MS) equipped with an HP-5 ms capillary column (30 m × 250 μm × 0.25 μm; J&W Scientific), operating in full scan mode over a mass range of 60 to 500 *m*/*z* under electron impact (EI) ionization at 70 eV. The GC oven temperature was initially set at 50 °C and held for 1.5 min, then ramped to 320 °C at 10 °C min^–1^ with a final hold for 3 min. Potential plasticizers were tentatively identified by comparing the acquired mass spectra with entries in the NIST library. To further confirm the identities of compounds with high spectral match scores, analytical standards of the corresponding plasticizers were purchased from Oakwood Products Inc., including di­(2-ethylhexyl) adipate (DOA, CAS: 103-23-1, 98%), DEHP (CAS: 117-81-7, 95%), and DOTP (CAS: 6422-86-2, 98%). Confirmation was achieved by comparing both the retention times and mass spectra of the hint compounds with those of the standards.

### 2.3 Leaching Experiments in Synthetic Aquatic Environments

The synthetic freshwater medium was prepared according to the recipe for artificial moderately hard freshwater.^39^ The seawater medium was prepared using commercial sea salt purchased from Instant Ocean.

A total of 50 mg of PVC MPs and 50 mL of synthetic freshwater or seawater medium was added to borosilicate glass vials sealed with aluminum foil-lined caps. Then, the vials underwent shaking using a rotisserie tube rotator at 80 rpm. Samples were collected at 0, 1, 2, 5, 10, and 20 d, and then filtered through 1 μm glass fiber filters (Sterlitech AE2500) to remove PVC MPs.

### 2.4 Leaching Experiments in a Fish In Vitro Digestion System

In the fish in vitro digestion experiment, gastric and intestinal buffers were prepared following the protocol described by Oldham et al.^34^ This protocol focuses on mimicking the physiological processes occurring in the stomach and the intestines. The main buffer used in this protocol was a modified Leibovitz’s L-15 cell culture medium. The main modification was the removal of amino acids and vitamins. Phosphate was also substituted with HEPES and bicarbonate in the intestinal buffer due to their physiological relevance. To simulate the gastric phase, 0.5 g of ground fish food (Supporting Information) and 1.45 mg of pepsin were added to 30 mL of gastric buffer, acidified with hydrochloric acid (pH = 2), maintaining an enzymatic activity of 12.5 U/mg of fish food. Then, 30 mg of PVC MPs were added to the mixture, vortexed briefly for 3–5 s, and immediately incubated at 37 °C on a shaker. After the 3 h gastric phase, an equal volume of intestinal buffer (1:1 v/v) was added to initiate the intestinal phase (pH = 8), followed by 3–5 s brief vortexing and continued incubation at 37 °C. The intestinal phase consisted of the modified L-15 buffer supplemented with 4 mg/mL bile and pancreatin, 20 mM HEPES, and 10 mM sodium bicarbonate. Samples were collected at: 0 (before PVC MPs addition), 0.1 (immediately after PVC MPs addition), 1, 3 (before intestinal buffer addition), 3.1 (immediately after intestinal buffer addition), 8, and 24 h. To evaluate the impact of food on additive leaching, parallel experimental groups were also prepared without the addition of fish food. All samples were filtered through 1 μm glass fiber filters (Sterlitech AE2500) to remove any remaining PVC MPs.

### 2.5 Quantification of Plasticizers

The aqueous samples were processed using solid-phase extraction (SPE) with C18 cartridges (Thermo Scientific) to selectively isolate the target compounds. The retained analytes were then eluted into acetonitrile at a 10:1 (v/v) ratio.^40^ The analysis of plasticizers in the solution was performed using a Shimadzu GC-2030/QP2020NX GC–MS system equipped with an Rtx-5 ms capillary column (30 m × 250 μm × 0.25 μm). The GC oven temperature was initially set at 40 °C, ramped to 260 °C at 20 °C min^–1^, and then held at 260 °C for 10 min. The ion source temperature was maintained at 200 °C. The mass spectrometer operated under EI ionization at 70 eV. Quantification was carried out in selected monitoring (SIM) mode. The specific ions used for SIM acquisition are listed in the Supporting Information. The hexane extracts of PVC MPs were also measured using a similar protocol to quantify the targeted additive contents in UV-aged and nonaged PVC MPs.

### 2.6 Quality Assurance and Quality Control

To minimize potential additives contamination, plastic instruments were avoided to the greatest extent. Glass syringes, stainless steel filter meshes, and borosilicate glass vials with aluminum foil-lined caps were employed throughout the experimental procedures. Solvent blanks and method blanks were included to monitor potential DEHP contamination from solvents, sample preparation, and instrumental analysis. Method blanks were processed using the same filtration, SPE extraction, elution, and GC–MS analysis procedures as experimental samples, but without PVC MPs. DEHP was consistently below the analytical LOD (8.21 μg L^–1^) in the method blanks, indicating no detectable background contamination. Quantification was performed using external calibration; isotopically labeled internal standards were not used. SPE recovery was evaluated by processing DEHP standards through the C18 SPE procedure. These recovery values were used to evaluate the extraction efficiency of the SPE step for DEHP in the processed liquid phase. Because this SPE recovery assessment did not include incubation, filtration, sorption to vial walls, or retention on PVC MPs or food particles, the reported recoveries should be interpreted as SPE recoveries rather than overall method recoveries or complete mass-balance recoveries. The limits of detection and quantification (LOD/LOQ) are also provided in the Supporting Information. All experiments were conducted in triplicate.

### 2.7 Statistical Analysis

Differences in MP size, the normalized O mass concentration, and ζ-potential were evaluated using a *t*-test. To evaluate leaching kinetics over time within individual treatments, DEHP concentrations were analyzed using a one-way analysis of variance (ANOVA) followed by Tukey’s post hoc test. All statistical tests were performed using Minitab software, with the level of significance set at α = 0.05.

## 3 Results and Discussion

### 3.1 UV-Aging of PVC MPs

SEM images showed cracks, pits, and surface roughening after UV exposure (Figure 1D). Despite these morphological alterations, particle size (Feret diameter) did not differ significantly between nonaged and UV-aged PVC MPs (*t*-test, *p* = 0.43). ATR-FTIR analysis (Figure 1F) revealed decreased signals in the C–H (3000–2800 cm^–1^), C=O (1760–1680 cm^–1^), and C–O (1310–1200 cm^–1^) regions, along with increased signals in regions associated with C=C (1660–1580 cm^–1^) and O–H (3550–3200 cm^–1^, 1400–1330 cm^–1^) groups. These spectral shifts are consistent with partial dehydrochlorination of the PVC polymer chains and suggest alterations in oxygen-containing functional groups, including possible formation of hydroxyl groups. EDS detected five main elements, C, O, Cl, Mg, and Al, in the PVC MPs (Supporting Information). The normalized O mass concentration increased significantly (*t*-test, *p* = 0.03) from 28.8 ± 2.9% to 33.0 ± 2.1% after aging. The presence of Mg and Al may be associated with inorganic additives or flame-retardant fillers commonly used in commercial wire insulation.^41^ The ζ-potential also significantly shifted from +5.9 ± 0.3 mV to −11.7 ± 0.5 mV after UV-aging (*t*-test, *p* = 0.01). This negative shift is consistent with increased surface oxidation after UV aging. Ionizable carboxyl groups may contribute to the more negative ζ-potential at near-neutral pH, while hydroxyl groups are more likely to contribute to increased surface hydrophilicity.

**Figure 1:**
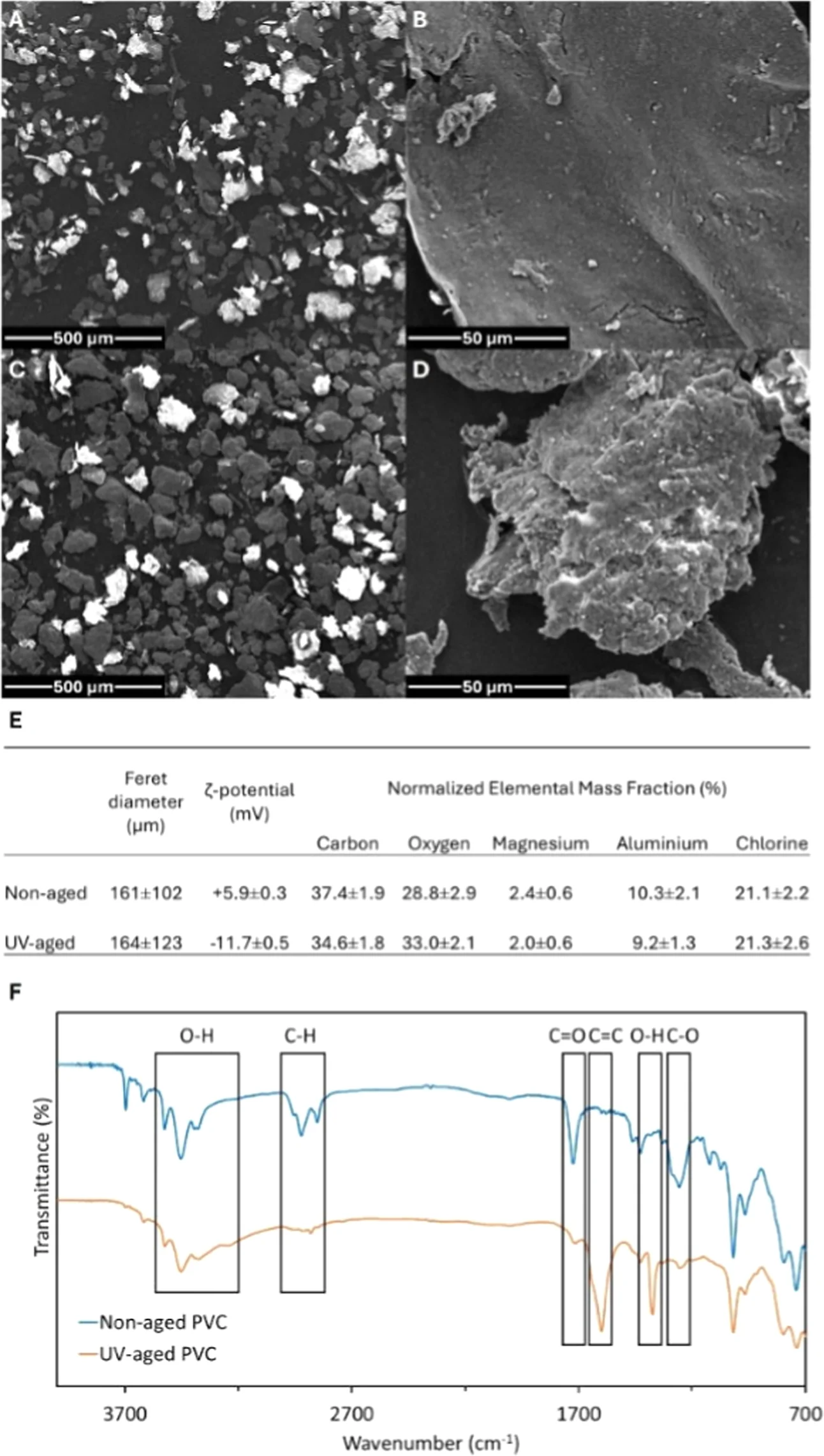
Characterization of the PVC MPs. (A,B) SEM micrograph of nonaged PVC MPs. (C,D) SEM micrograph of UV-aged PVC MPs. (E) Feret diameter, ζ-potential, and normalized elemental mass fractions of nonaged and UV-aged PVC MPs. (F) ATR-FTIR spectra of nonaged and UV-aged PVC MPs.

The photodegradation of PVC polymer generally initiates with dehydrochlorination, leading to the formation of alkene and polyene structures.^42^ Under oxic conditions, molecular oxygen can react with these conjugated double bonds, yielding α, β-unsaturated ketones and alcohols.^42^ However, in commercial PVC products containing various additives, the changes on ATR-FTIR spectra likely reflect not only polymer degradation but also the breakdown of these additives. Plasticizers such as phthalates are susceptible under UV exposure. This multistep process typically yields intermediate products including phthalate monoesters, phthalaldehyde, phthalic anhydride, and phthalic acid, which can be further oxidized into CO_2_ and water.^43^ Thus, the loss of C=O and C–O signals observed in ATR-FTIR spectra may partially reflect the progressive photodegradation of plasticizers in the PVC MPs.

It should be noted that the UV-aging treatment used in this study was intended as an accelerated aging approach rather than a direct simulation of natural solar exposure. The 254 nm irradiation used here has a photon energy of approximately 471 kJ mol^–1^, which is higher than that of UVB (280–315 nm; approximately 380–427 kJ mol^–1^) and UVA (315–400 nm; approximately 299–380 kJ mol^–1^) radiation that dominate environmentally relevant sunlight. The photon energy of 254 nm irradiation is within or above the range of bond dissociation energies for several bonds relevant to PVC and phthalate plasticizers, particularly C–Cl and some C–C and C–O bonds. Therefore, this treatment can accelerate PVC dehydrochlorination, chain scission, surface oxidation, and plasticizer photolysis. However, it may also produce aging pathways and additive mobility patterns that differ from those generated under natural aging conditions. Thus, our present results only demonstrate the potential effect of severe photoaging on DEHP mobility, but should not be interpreted as quantitatively representative of natural aging rates.

### 3.2 Additives Identification

From the chromatographic spectrum of the hexane extract of nonaged PVC MPs, we tentatively identified five additives based on the NIST library, and their corresponding mass spectra are provided in the Supporting Information. By matching their retention times with those of purchased analytical standards, we further confirmed DOA, DEHP, and DOTP among these compounds. Their amounts recovered in the hexane extract corresponded to 0.0070%, 0.41%, and 0.91% of the mass of nonaged PVC MPs, and 0.0021%, 0.098%, and 0.17% of the mass of UV-aged PVC MPs, respectively (w/w, Supporting Information). The lower extractable plasticizer contents in UV-aged samples indicate degradation of these plasticizers during UV-aging treatment, consistent with the ATR-FTIR spectra. The remaining compounds showed high matching scores with diundecyl phthalate (DUP, CAS: 3648-20-2) and di­(2-ethylhexyl) tetrabromophthalate (TBPH, CAS: 26040-51-7) in the NIST library. However, due to the unavailability of commercial analytical standards, we were not able to further confirm the identities of these compounds. In the full scan of UV-aged PVC extract, several minor peaks indicate potential degradation products formed during UV-aging (mass spectra, Supporting Information). However, most of these peaks exhibited low matching scores or lacked reliable identification in the NIST library.

Although DOTP showed a higher hexane-extractable content than DEHP, we selected DEHP as the target compound for subsequent leaching experiments. First, analytical standards were available for DEHP, allowing retention time confirmation and quantitative analysis. Second, DEHP was the dominant quantifiable compound observed in the leaching samples during the kinetic experiments, whereas DOA and DOTP produced weak or unquantifiable signals under the present aqueous and digestion conditions. This likely reflects differences in physicochemical properties, including hydrophobicity, aqueous solubility, and partitioning behavior, rather than only their initial abundance in the PVC material. Third, DEHP remains one of the most widely studied phthalate plasticizers and has strong environmental and regulatory relevance due to its persistence, widespread occurrence, and endocrine-disrupting potential. Therefore, DEHP was used as a representative target plasticizer in this study. However, the conclusions drawn here should be interpreted specifically for DEHP and should not be directly generalized to other plasticizers, such as DOA or DOTP, which may differ in leaching kinetics, transformation pathways, and environmental mobility.

The detected additives also reflect the increasing diversity of plasticizers used in commercial PVC materials. Traditional phthalates, such as DEHP, have been widely used because of their high compatibility and plasticizing efficiency in PVC.^44^ However, because of concerns regarding their adverse health effects, the European Union began phasing out DEHP and several other major phthalates in 2008.^45,46^ In response, manufacturers have developed and used a variety of alternative plasticizers, including adipates, benzoates, citrates, cyclohexane dicarboxylates, phosphate esters, and terephthalates,^47^ represented in this study by compounds such as DOA and DOTP. The rapid development and diversification of plastic additives create analytical challenges, as many newly introduced, proprietary, or transformation products are not included in existing mass spectral libraries, and corresponding analytical standards are often unavailable. These limitations make it difficult to confirm their identities and accurately quantify their presence in complex plastic samples.

### 3.3 DEHP Leaching in Synthetic Environmentally Relevant Aqueous Media

We evaluated DEHP leaching from nonaged and UV-aged PVC MPs in freshwater and seawater. Overall, UV-aged PVC MPs leached significantly more DEHP than nonaged MPs in both media, with consistently higher apparent DEHP concentrations observed in freshwater than in seawater (Figure 2, *t*-test, *p* < 0.01 at time points after *t* = 0). In nonaged PVC MPs groups, DEHP appeared only in seawater after 20 d, but its apparent concentration remained below the LOQ (24.49 μg L^–1^), and no DEHP was detected in freshwater. In contrast, UV-aged PVC MPs leached measurable amounts of DEHP in both freshwater and seawater throughout the experiment. The leaching kinetics from UV-aged PVC MPs followed a similar pattern in both media: apparent concentrations peaked on day 1, then remained stable from day 5 to day 20 with no statistically significant differences observed (Tukey test with 95% confidence). In freshwater, the peak apparent concentration on day 1 was 105.6 ± 2.2 μg L^–1^ (or 105.6 ± 2.2 μg g^–1^ MPs), with a stabilized apparent concentration of 63.1 ± 4.4 μg L^–1^ (or 63.1 ± 4.4 μg g^–1^ MPs); while in seawater, the corresponding values were 70.8 ± 2.0 μg L^–1^ (or 70.8 ± 2.0 μg g^–1^ MPs) and 46.8 ± 3.6 μg L^–1^ (or 46.8 ± 3.6 μg g^–1^ MPs), respectively.

**Figure 2:**
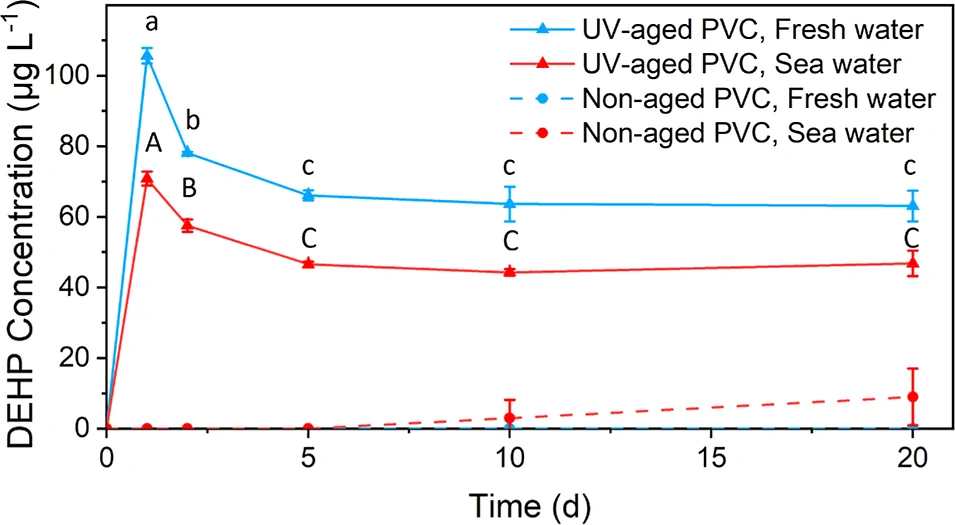
Time-dependent apparent concentrations of di­(2-ethylhexyl) phthalate (DEHP) in the filtered aqueous phase during leaching from polyvinyl chloride microplastics (PVC MPs) in synthetic freshwater and seawater over a 20 day period. The particle concentration was maintained at 1 mg mL^–1^. Four experimental groups are included: nonaged MPs in freshwater, UV-aged MPs in freshwater, nonaged MPs in seawater, and UV-aged MPs in seawater. Error bars represent the standard deviation (*n* = 3). Lowercase letters (a, b, c) and uppercase letters (A, B, C) denote Tukey grouping of DEHP concentrations over time from UV-aged PVC MPs in fresh water and in seawater, respectively (Tukey test with 95% confidence).

The solubility of DEHP in water has been reported to vary widely, ranging from 1.9 to 360 μg L^–1^ at 20–25 °C across different studies.^48^ Under ideal equilibrium conditions, the thermodynamic solubility of the freely dissolved fraction is reported as 3.6 μg L^–1^ at 20 °C.^23^ However, the values reported in the present study should be interpreted as operationally defined apparent concentrations rather than strict representations of the freely dissolved DEHP fraction or a complete mass balance. Specifically, they represent the DEHP fraction that passed the 1 μm filtration step, was extracted by C18 SPE, and was analyzed by GC–MS. The apparent concentrations may include freely dissolved DEHP as well as colloid-associated, microdroplet-associated, dissolved-organic-matter-associated, or potentially nanoplastic-associated DEHP that passed the filter. Consequently, the apparent concentrations may exceed the thermodynamic solubility of freely dissolved DEHP. Because the method was not designed to separate these fractions, it cannot distinguish freely dissolved DEHP from nonfreely dissolved fractions.

Previous research indicates that aging treatments increase particle surface hydrophilicity and enlarged the surface area,^38,49^ while breaking down polymer chains, which promotes internal migration of additives within the particles.^50^ In our study, these changes enhanced the accumulation of DEHP on MPs surfaces, leading to higher leaching from UV-aged MPs compared with nonaged MPs. Although aging also increased surface roughness and decreased crystallinity, which could enhance phthalate adsorption, these effects were minor relative to partitioning and hydrophobic interactions.^24^

The higher leaching of DEHP in freshwater compared to seawater can be attributed to the higher salinity of seawater. Increased ionic strength enhances water–ion interactions and reduces water’s ability to solvate hydrophobic molecules, thereby decreasing the freely dissolved DEHP fraction. In addition, increased ionic strength can further reduce apparent aqueous DEHP concentrations by destabilizing colloidal or microdroplet-associated DEHP, which enhances its partitioning to solid phases.

The leaching of additives from MPs generally involves two stages: instantaneous leaching and continuous leaching.^23,49^ Instantaneous leaching represents the rapid desorption of additives bound to the particle surface immediately upon water contact, followed by continuous leaching, which is much slower and time dependent. In this study, the sharp rise in apparent DEHP concentration on day 1 reflects instantaneous leaching, after which the process transitioned into continuous leaching. The decrease in apparent concentration from day 1 to day 5 is likely resulting from the partitioning of DEHP to the vial walls as the system approaches equilibrium. The higher apparent DEHP concentration from UV-aged PVC MPs compared to nonaged PVC, particularly during the instantaneous leaching phase, suggests that UV treatment promotes the accumulation of DEHP on the particle surface. During continuous leaching, DEHP leaching was more strongly influenced by water salinity and agitation than by particle size, temperature, or pH, owing to the high hydrophobicity of phthalates.^25,51^

In natural environments, phthalates also undergo degradation, primarily through biodegradation, hydrolysis, and photolysis. Among these, biodegradation generally dominates in most media.^52^ The hydrolysis rate of phthalates is strongly influenced by pH and temperature. At neutral pH, hydrolysis is negligible, with reported half-lives ranging from 3.2 years for DMP to nearly 2000 years for DEHP, whereas acidic or alkaline conditions significantly accelerate hydrolysis.^53^ Photolysis also contributes to phthalate removal in aquatic environments, with half-lives estimated at 2.4–12 years for DEP and DBP and 0.12–1.5 years for DEHP.^53^ Phthalates in river sediments reportedly degrade with average half-lives of 2.5–14.8 days under aerobic conditions and 14.4–34.7 days under anaerobic conditions.^54^ In our study, the decline of DEHP from day 1 to day 5 is unlikely to have resulted from biodegradation, hydrolysis, or photolysis, since the laboratory prepared solution likely lacked the corresponding microorganisms and no further concentration decreases were observed afterward. Instead, this reduction may reflect reabsorption onto PVC MPs due to the high hydrophobicity and low solubility of phthalates.^24^

In natural and engineered water systems, the fate of DEHP leached from PVC MPs should be interpreted as a combination of partitioning and transformation processes rather than as simple accumulation in the water phase. Because DEHP is highly hydrophobic, surrounding sink phases that were not included in the present synthetic media, such as natural organic matter (NOM), suspended solids, sediments, biofilms, and activated sludge can provide additional sorption domains for hydrophobic plasticizers.^25^ These processes may reduce the freely dissolved DEHP fraction while increasing its association with particulate or organic-rich phases. Biofilms may further modify MP surface properties and create local microenvironments that influence additive leaching, sorption, and degradation.^55^

Therefore, in natural systems, PVC MPs may contribute more to localized transfer of DEHP to adjacent sink phases, particularly sediments and organic matter, than to sustained accumulation in the bulk aqueous phase. Similar processes are relevant in wastewater treatment plants (WWTPs), where activated sludge and suspended organic solids may promote the partitioning of DEHP into biosolids and reduce its freely dissolved fraction in the aqueous effluent.^56^ This interpretation is consistent with previous observations of phthalate accumulation in WWTP sludge and with studies identifying wastewater effluent, surface runoff, drainage, and atmospheric deposition as major sources of phthalate pollution in aquatic environments.^57^ In drinking water systems, particle removal processes and relatively low MP abundance would likely limit direct aqueous exposure from MP-derived DEHP, although aged plastic particles may still contribute to localized additive transfer where particles, organic matter, and biofilms coexist. In parallel with partitioning, DEHP may also undergo transformation through biodegradation, photolysis, and, to a lesser extent under neutral conditions, hydrolysis, generating products such as mono­(2-ethylhexyl) phthalate (MEHP), 2-ethylhexanol, phthalic acid derivatives, and smaller oxidation products. However, because the present study quantified DEHP only and did not target transformation products, the observed concentration profiles should be interpreted as DEHP leaching and redistribution rather than a complete description of DEHP fate. Future work should combine leaching experiments with targeted or nontarget analysis of transformation products to better resolve the chemical fate of plasticizer-derived compounds after release from aged PVC MPs.

### 3.4 DEHP Leaching in an In Vitro Digestion System

Similar to the leaching experiments in synthetic freshwater and seawater, we targeted the DEHP in the in vitro digestion system. Only UV-aged PVC MPs leached detectable amounts of DEHP (Figure 3). In this study, we use bioaccessibility to refer to the DEHP fraction present in the filtered digestive-fluid phase after in vitro digestion and recoverable by the analytical workflow; it should not be interpreted as bioavailability because intestinal uptake, metabolism, and tissue accumulation were not measured. In the gastric phase, apparent DEHP concentrations increased rapidly within the first hour due to instantaneous leaching, stabilizing at 27.9 ± 2.3 μg L^–1^ and 50.0 ± 3.4 μg L^–1^ from 1 to 3 h in the with-food and without-food groups, respectively (Tukey test with 95% confidence). Immediately after intestinal buffer was added at 3 h, the apparent DEHP concentration in both groups dropped to 20.5 ± 3.5 μg L^–1^ and 28.2 ± 1.3 μg L^–1^, respectively, but subsequently increased and stabilized at 27.3 ± 2.0 μg L^–1^ and 49.8 ± 2.4 μg L^–1^ from 8 to 24 h (Tukey test, 95% confidence Figure 3), suggesting re-equilibration under the intestinal conditions. The faster approach to steady state compared with the aquatic leaching experiments likely results from enhanced mass transfer during brief vortexing after MPs and intestinal buffer additions, as well as the smaller working volume (30 mL vs 50 mL).

**Figure 3:**
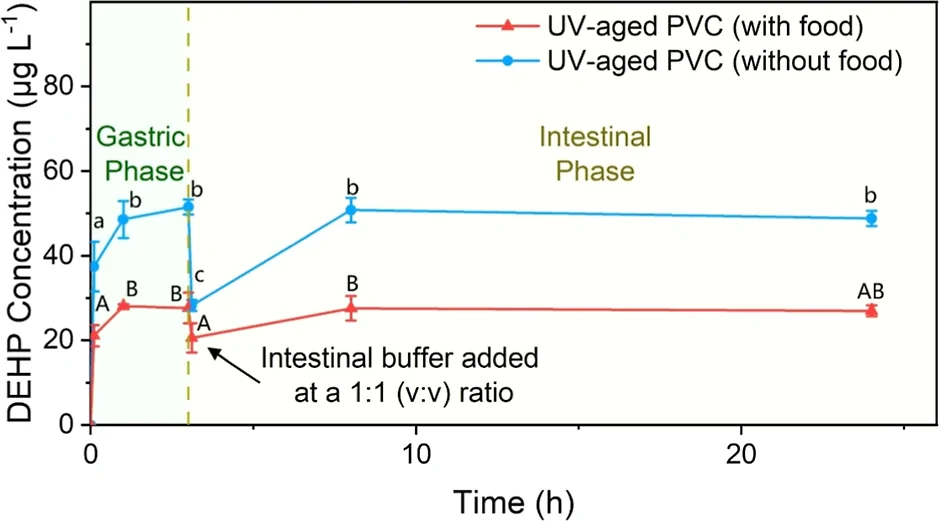
Time-dependent apparent concentrations of di­(2-ethylhexyl) phthalate (DEHP) in the filtered digestive-fluid phase during fish in vitro digestion models containing 1 mg mL^–1^ of polyvinyl chloride microplastics (PVC MPs). PVC MPs were first incubated in gastric buffer for 3 h, after which intestinal buffer was added at a 1:1 volume ratio to simulate the intestinal phase from 3 to 24 h, maintaining 37 °C throughout. Error bars represent one standard deviation (*n* = 3). Uppercase letters (A, B) and lowercase letters (a, b) denote Tukey grouping of DEHP concentrations over time from UV-aged PVC MPs with and without adding ground fish food, respectively (Tukey test with 95% confidence).

Previous studies have demonstrated that food and coingested sediment can reduce the bioavailability of hydrophobic additives in the digestive system, as these compounds tend to sorb onto indigestible particles, particularly undigested proteins and dietary fiber.^58,59^ In our study, apparent DEHP concentrations were lower in the with-food group compared to the without-food group, suggesting that food particles limited the operationally defined bioaccessible DEHP fraction in the digestive-fluid phase. Although some studies have reported higher bioavailability under fed state conditions compared to the fasted state;^60,61^ however, these studies simulated these two states only by altering the recipe of the digestive fluids, but without adding food, which may overlook the potential for additives to interact with food matrices. A more likely explanation is that the fed state initially promotes the leaching of phthalates from the MPs; however, these leached phthalates are rapidly sorbed by food particles. At equilibrium, phthalates distribute among the MPs, food particles, and digestive fluids. Since part of the phthalates remains adsorbed onto the MPs and food particles, their partitioning into the digestive fluids is reduced. We should also note that the present set up does not include intestinal epithelium. Intestinal cells may also sorb DEHP, thereby reducing the apparent DEHP concentration in the digestive-fluid phase and potentially driving further leaching.^62,63^

The rapid increase in apparent DEHP concentration in the filtered digestive-fluid phase after adding the intestinal buffer is also noteworthy. This trend likely reflects a re-equilibration of DEHP between the particle surface and the fluid phase. Because phthalates are highly hydrophobic with low solubility,^64^ a substantial fraction of DEHP is inhibited from leaching into digestive fluids and is expected to remain adsorbed onto MPs. Previous studies have reported that PVC MPs can continue to leach DBP even after ten consecutive leaching cycles,^24^ suggesting that digestive secretions may further promote the leaching of phthalates during digestion.

The pharmacokinetics of phthalates are determined by the processes of protein binding, ionization, passive partitioning, and metabolism, with passive partitioning influencing all these processes.^65^ In addition to being adsorbed onto MPs and food particles, phthalates in the digestive system can also bind to digestive enzymes such as pepsin and trypsin through hydrophobic interactions, hydrogen bonding, and π–π interactions.^66,67^ In the intestinal phase, enzymes such as esterases and lipases can hydrolyze DEHP into monoesters such as MEHP.^68^ Previous studies also suggest that fecal microbiota can act as both sorptive and metabolic systems, further promoting plasticizer leaching and hydrolysis.^69^ The fate of DEHP in digestive fluids should therefore be interpreted as a dynamic balance among leaching, partitioning, binding, and potential transformation. At the same time, DEHP can partition among PVC MPs, food particles, digestive fluids, bile-associated phases, and proteins or enzymes. The relatively stable apparent DEHP concentrations observed from 8 to 24 h therefore should not be interpreted as evidence of chemical stability alone. Instead, they may reflect a pseudosteady state in which DEHP released from UV-aged PVC MPs is balanced by sorption to food particles or biomolecules, repartitioning to MPs, and possible enzymatic transformation. Because this study quantified only DEHP and did not measure MEHP or other transformation products, the data cannot resolve the full chemical fate of DEHP after leaching. Nevertheless, these processes suggest that digestive conditions may maintain concentration gradients that promote continued additive release while simultaneously transforming or redistributing the released plasticizer. Future studies combining in vitro digestion with targeted analysis of DEHP metabolites and nontarget screening would be needed to evaluate these transformation pathways directly.

The retention time of MPs in fish digestive systems depends on particle size and the digestive tract structure. In general, retention time decreases as particle size increases.^70,71^ In fish species with a stomach, larger particles tend to be actively evacuated more rapidly whereas stomachless species primarily rely on passive excretion of MPs along with the chyme.^71^ In most cases, retention time is within 24 h,^71^ thus, the leaching of DEHP is supposed to be mainly dominated by instantaneous leaching and re-equilibration induced by digestive secretions. In addition, MPs smaller than 5 μm are considered capable of crossing an intact gastrointestinal barrier and persisting in fish tissues or organs, with translocation rates increasing as particle size decreases.^72^ However, MPs with much larger sizes (501–1500 μm) have also been reported in muscle tissue.^73^ Together, the detection of the MPs in fish tissues or organs highlights the need to consider continuous DEHP leaching in risk evaluations.

### 3.5 Environmental Relevance and Limitations

Environmental comparison of MP abundance is complicated by differences in reporting units. Most field studies report MP abundance as particle number per unit volume or sediment mass. For example, high reported values include 88 particles per liter in surface waters and 38,790 particles per kilogram in sediments.^74^ In the present study, we used a mass-based MP loading of 1 mg mL^–1^ (1000 mg L^–1^). Direct conversion between particle number and mass concentration depends strongly on particle size and density. For context, reported mass-based MP concentrations in seawater include approximately 0.003–0.023 mg L^–1^ and, in some cases, up to 4.5 mg L^–1^.^75^ This high MP loading was selected to ensure that leached DEHP exceeded the instrumental LOQ. Therefore, our closed-vial system should be interpreted as a controlled mechanistic probe designed to isolate leaching behaviors, rather than as a direct simulation of environmental MP exposure. In addition, the 254 nm UV-aging treatment represents an accelerated photoaging condition rather than natural solar aging; therefore, the extent of PVC oxidation, plasticizer degradation, and subsequent DEHP leaching may differ under environmentally relevant sunlight exposure.

These methodological constraints can alter partitioning behavior. In a closed-vial system with a high MP-to-water ratio, the apparent DEHP concentration may rapidly approach a steady state controlled by hydrophobic partitioning among the aqueous phase, PVC MPs, vial walls, and nonfreely dissolved DEHP fractions. Thus, the concentrations measured in this study should not be regarded as strictly representing the freely dissolved DEHP fraction.

Under realistic conditions, however, MP abundances are much lower, and aqueous concentrations of leached DEHP would generally be expected to remain low. Thus, the surrounding water, sediments, dissolved organic matter, suspended particles, and biota may act as larger sink phases, helping to maintain a concentration gradient and promoting continued leaching of the readily mobile DEHP fraction from PVC MPs. At the same time, environmental processes such as dilution, advection, biodegradation, photolysis, and sorption to organic-rich sediments would limit the accumulation of DEHP in natural waters. Therefore, the results should not be used to directly estimate environmental DEHP concentrations from PVC MPs. Instead, they demonstrate that UV aging can increase the availability of mobile DEHP fractions and enhance initial leaching when aged PVC MPs encounter new aqueous or digestive environments.

Similar caution is needed when interpreting the fish in vitro digestion results. A global review found that while 49% of fish sampled contained MPs, the burden averaged 3.5 pieces per individual.^76^ This relatively low particle burden suggests that phthalate leaching from ingested MPs is less likely to represent a dominant exposure pathway at the whole-organism level. Dietary exposure, ambient water, sediment-associated phthalates, and prey items may be more important sources, especially for benthic and demersal species.^77^

Nevertheless, the digestion experiments provide mechanistic evidence that UV aging and food matrices influence the bioaccessible fraction of DEHP leached from PVC MPs. These results are relevant for understanding short-term leaching under digestive conditions, but they should not be extrapolated to organism-level exposure without considering realistic particle burdens, gut residence time, intestinal uptake, metabolism, and other exposure routes. Importantly, given recent findings reporting considerable numbers of particles in the fillets and livers of many surveyed fish,^78^ such internal accumulation may involve longer exposure durations and distinct toxicological mechanisms, warranting further investigation.

## 4 Environmental Implications

Aging influences the leaching of additives from MPs. As plastics naturally age, they undergo physical and chemical changes, including enlarged surface area, increased surface hydrophilicity, and partial breakdown of polymer chains. These changes can facilitate the migration of additives from the particle interior to the surface, making them available for leaching, especially when the particles enter new aquatic environments. This study further shows that DEHP leaching is matrix-dependent: elevated salinity reduced apparent DEHP concentrations, while ground fish food acted as a sorptive matrix and reduced the bioaccessible DEHP fraction in digestive fluids. These findings indicate that the fate of MP-associated plasticizers depends not only on polymer aging state, but also on the surrounding environmental or physiological matrix. However, the present experiments should be interpreted as a mechanistic probe rather than as direct estimates of environmental concentrations under realistic MP abundances. Future assessments should account for MP aging state, environmental matrix, and the distinction between bioaccessible fractions and the freely dissolved fraction.

## Supplementary Material



## Notes

Fish food ingredients, identification of plasticizers in MPs in GCMS; total ion chromatogram (TIC) of nonaged PVC MPs; main peaks mass spectrum and the hint compounds; DEHA, DEHP, and DOTP physicochemical properties, quantifier/qualifier ions, and analytical limit; leached additives relative to PVC mass; EDS spectrum of PVC MPs, and mass spectra of likely degradation products of plasticizers (PDF)

## References

[1] Ciacci L., Passarini F., Vassura I. (2017). The European PVC Cycle: In-Use Stock
and Flows. Resour., Conserv. Recycl..

[2] Liu Y., Zhou C., Li F., Liu H., Yang J. (2020). Stocks and
Flows of Polyvinyl Chloride (PVC) in China. Resour., Conserv. Recycl..

[3] Godwin, A. D. 26—Plasticizers. In Applied Plastics Engineering Handbook, 3rd ed; Kutz, M. , Ed.; Plastics Design Library; William Andrew Publishing, 2024; pp 595–618.10.1016/B978-0-323-88667-3.00031-X.

[4] Hennebert P. (2021). Hazardous
Properties of Brominated, Phosphorus, Chlorinated, Nitrogen and Mineral
Flame Retardants in Plastics Which May Hinder Their Recycling. Detritus.

[5] Chen Y., Zhou S., Pan S., Zhao D., Wei J., Zhao M., Fan H. (2022). Methods for
Determination of Plasticizer
Migration from Polyvinyl Chloride Synthetic Materials: A Mini Review. J. Leather Sci. Eng..

[6] Gao C., Wang Y., Gao Y., Wang R., Wang H., Wu Y., Liu Y. (2022). Structure
Design, Performance Simulation and Plasticizing
Properties of Bio-Based Copolyesters Plasticizer on PVC. J. Polym. Environ..

[7] Wang T., Kanda H., Kusumi K., Mei L., Zhang L., Machida H., Norinaga K., Yamamoto T., Sekikawa H., Yasui K., Zhu L. (2024). Environmental-Friendly
Extraction
of Di­(2-Ethylhexyl) Phthalate from Poly­(Vinyl Chloride) Using Liquefied
Dimethyl Ether. Waste Manag..

[8] Coltro L., Pitta J. B., da Costa P. A., Fávaro
Perez M. Â., de Araújo V. A., Rodrigues R. (2014). Migration
of Conventional and New Plasticizers from PVC Films into Food Simulants:
A Comparative Study. Food Control.

[9] Marcilla A., García S., García-Quesada J. C. (2004). Study of
the Migration of PVC Plasticizers. J. Anal.
Appl. Pyrolysis.

[10] Alp A. C., Yerlikaya P. (2020). Phthalate Ester Migration into Food:
Effect of Packaging
Material and Time. Eur. Food Res. Technol..

[11] Babich M. A., Bevington C., Dreyfus M. A. (2020). Plasticizer Migration from Children’s
Toys, Child Care Articles, Art Materials, and School Supplies. Regul. Toxicol. Pharmacol..

[12] Kastner J., Cooper D. G., Marić M., Dodd P., Yargeau V. (2012). Aqueous Leaching
of Di-2-Ethylhexyl Phthalate and “Green” Plasticizers
from Poly­(Vinyl Chloride). Sci. Total Environ..

[13] Ong H.-T., Samsudin H., Soto-Valdez H. (2022). Migration
of Endocrine-Disrupting
Chemicals into Food from Plastic Packaging Materials: An Overview
of Chemical Risk Assessment, Techniques to Monitor Migration, and
International Regulations. Crit. Rev. Food Sci.
Nutr..

[14] Panneel L., Malarvannan G., Jorens P. G., Covaci A., Mulder A. (2022). Plasticizers
in the Neonatal Intensive Care Unit: A Review on Exposure Sources
and Health Hazards. Crit. Rev. Environ. Sci.
Technol..

[15] Lu L., Li W., Cheng Y., Liu M. (2023). Chemical Recycling Technologies for
PVC Waste and PVC-Containing Plastic Waste: A Review. Waste Manag..

[16] Zakharyan E. M., Petrukhina N. N., Maksimov A. L. (2020). Pathways of Chemical Recycling of
Polyvinyl Chloride: Part 1. Russ. J. Appl. Chem..

[17] Wang C., Zhao J., Xing B. (2021). Environmental
Source, Fate, and Toxicity
of Microplastics. J. Hazard. Mater..

[18] Hess K. Z., Forsythe K., Wang X., Tipling G., Jones J., Mata M., Hughes V., Martin C., Doyle J., Scott J., Minghetti M., Jilling A., Cerrato J. M., El Hayek E., Gonzalez-Estrella J. (2024). Open dumping
and burning: an overlooked
source of terrestrial microplastics in underserved communities. ChemRxiv.

[19] Tumwesigye E., Felicitas Nnadozie C., C Akamagwuna F., Siwe Noundou X., William Nyakairu G., Odume O. N. (2023). Microplastics as
Vectors of Chemical
Contaminants and Biological Agents in Freshwater Ecosystems: Current
Knowledge Status and Future Perspectives. Environ.
Pollut..

[20] Liu H., Zhao M., Jiang F. (2025). Aging of PVC
Pipes Induces Release
of Microplastics: Insight into the Role of the Hydrophilicity of Pipe
Inner Surface. Process Saf. Environ. Prot..

[21] Mintenig S. M., Löder M. G. J., Primpke S., Gerdts G. (2019). Low Numbers
of Microplastics
Detected in Drinking Water from Ground Water Sources. Sci. Total Environ..

[22] Ben
Stride, Abolfathi S., Bending G. D., Pearson J. (2024). Quantifying Microplastic Dispersion
Due to Density Effects. J. Hazard. Mater..

[23] Henkel C., Hüffer T., Hofmann T. (2022). Polyvinyl Chloride
Microplastics
Leach Phthalates into the Aquatic Environment over Decades. Environ. Sci. Technol..

[24] Yan Y., Zhu F., Zhu C., Chen Z., Liu S., Wang C., Gu C. (2021). Dibutyl Phthalate Release from Polyvinyl Chloride Microplastics:
Influence of Plastic Properties and Environmental Factors. Water Res..

[25] Henkel C., Lamprecht J., Hüffer T., Hofmann T. (2023). Environmental Factors
Strongly Influence the Leaching of Di­(2-Ethylhexyl) Phthalate from
Polyvinyl Chloride Microplastics. Water Res..

[26] Ye X., Wang P., Wu Y., Zhou Y., Sheng Y., Lao K. (2020). Microplastic Acts as a Vector for Contaminants: The Release Behavior
of Dibutyl Phthalate from Polyvinyl Chloride Pipe Fragments in Water
Phase. Environ. Sci. Pollut. Res..

[27] Ding J., Sun C., He C., Li J., Ju P., Li F. (2021). Microplastics
in Four Bivalve Species and Basis for Using Bivalves as Bioindicators
of Microplastic Pollution. Sci. Total Environ..

[28] Suckling C. C. (2021). Responses
to Environmentally Relevant Microplastics Are Species-Specific with
Dietary Habit as a Potential Sensitivity Indicator. Sci. Total Environ..

[29] Yap V. H. S., Chase Z., Wright J. T., Hurd C. L., Lavers J. L., Lenz M. (2020). A Comparison with Natural
Particles Reveals a Small Specific Effect
of PVC Microplastics on Mussel Performance. Mar. Pollut. Bull..

[30] Barboza L. G. A., Lopes C., Oliveira P., Bessa F., Otero V., Henriques B., Raimundo J., Caetano M., Vale C., Guilhermino L. (2020). Microplastics
in Wild Fish from North East Atlantic
Ocean and Its Potential for Causing Neurotoxic Effects, Lipid Oxidative
Damage, and Human Health Risks Associated with Ingestion Exposure. Sci. Total Environ..

[31] Li B., Liang W., Liu Q.-X., Fu S., Ma C., Chen Q., Su L., Craig N. J., Shi H. (2021). Fish Ingest
Microplastics Unintentionally. Environ. Sci.
Technol..

[32] Rios-Fuster B., Alomar C., Paniagua González G., Garcinuño
Martínez R. M., Soliz Rojas D. L., Fernández Hernando P., Deudero S. (2022). Assessing Microplastic Ingestion and Occurrence of
Bisphenols and Phthalates in Bivalves, Fish and Holothurians from
a Mediterranean Marine Protected Area. Environ.
Res..

[33] Peng Y., Lu J., Fan L., Dong W., Jiang M. (2024). Simulated Gastrointestinal
Digestion of Two Different Sources of Biodegradable Microplastics
and the Influence on Gut Microbiota. Food Chem.
Toxicol..

[34] Oldham D., Dudefoi W., Minghetti M. (2023). Development
of an in Vitro Digestion
System to Study the Bioavailability and Bioreactivity of Zinc Sulfate
and Zn-Bioplex in Fish Using the RTgutGC Cell Line. ACS Food Sci. Technol..

[35] Peters R., de Jong N., de Haan L., Wright S., Bouwmeester H. (2022). Release and
Intestinal Translocation of Chemicals Associated with Microplastics
in an in Vitro Human Gastrointestinal Digestion Model. Microplast. Nanoplast..

[36] Wu P., Tang Y., Jin H., Song Y., Liu Y., Cai Z. (2020). Consequential Fate
of Bisphenol-Attached PVC Microplastics in Water
and Simulated Intestinal Fluids. Environ. Sci.
Ecotechnology.

[37] Masset T., Ferrari B. J. D., Oldham D., Dudefoi W., Minghetti M., Schirmer K., Bergmann A., Vermeirssen E., Breider F. (2021). In Vitro Digestion of Tire Particles
in a Fish Model
(Oncorhynchus Mykiss): Solubilization Kinetics of Heavy Metals and
Effects of Food Coingestion. Environ. Sci. Technol..

[38] Cai R., Scott J., Thennakoon D., Materer N., Minghetti M., Gonzalez-Estrella J. (2024). Adsorption
of Alachlor, Lindane, and Methomyl onto
Polystyrene Microplastics: Effects of Aging Treatments. Environ. Eng. Sci..

[39] US Environmental Protection Agency Methods for Measuring the Acute Toxicity of Effluents and Receiving Waters to Freshwater and Marine Organisms, 5th ed.; Washington, DC, 2002.

[40] Russo M. V., Avino P., Perugini L., Notardonato I. (2015). Extraction
and GC-MS Analysis of Phthalate Esters in Food Matrices: A Review. RSC Adv..

[41] Rothon, R. ; Hornsby, P. Chapter 9—Fire Retardant Fillers for Polymers. In Polymer Green Flame Retardants; Papaspyrides, C. D. , Kiliaris, P. , Eds.; Elsevier: Amsterdam, 2014; pp 289–321.10.1016/B978-0-444-53808-6.00009-3.

[42] Wang C., Xian Z., Jin X., Liang S., Chen Z., Pan B., Wu B., Ok Y. S., Gu C. (2020). Photo-Aging of Polyvinyl
Chloride Microplastic in the Presence of Natural Organic Acids. Water Res..

[43] Hankett J. M., Collin W. R., Chen Z. (2013). Molecular
Structural Changes of Plasticized
PVC after UV Light Exposure. J. Phys. Chem.
B.

[44] Rahman M., Brazel C. S. (2004). The Plasticizer
Market: An Assessment of Traditional
Plasticizers and Research Trends to Meet New Challenges. Prog. Polym. Sci..

[45] Tickner J. A., Schettler T., Guidotti T., McCally M., Rossi M. (2001). Health Risks
Posed by Use of Di-2-Ethylhexyl Phthalate (DEHP) in PVC Medical Devices:
A Critical Review. Am. J. Ind. Med..

[46] Petersen J. H., Jensen L. K. (2010). Phthalates and Food-Contact
Materials: Enforcing the
2008 European Union Plastics Legislation. Food
Addit. Contam. Part A.

[47] Jung J., Cho Y., Lee Y., Choi K. (2024). Uses and Occurrences
of Five Major
Alternative Plasticizers, and Their Exposure and Related Endocrine
Outcomes in Humans: A Systematic Review. Crit.
Rev. Environ. Sci. Technol..

[48] Li L., Zhang Z., Men Y., Baskaran S., Sangion A., Wang S., Arnot J. A., Wania F. (2022). Retrieval, Selection,
and Evaluation of Chemical Property Data for Assessments of Chemical
Emissions, Fate, Hazard, Exposure, and Risks. ACS Environ. Au.

[49] Henkel C., Hüffer T., Peng R., Gao X., Ghoshal S., Hofmann T. (2024). Photoaging Enhances the Leaching
of Di­(2-Ethylhexyl)
Phthalate and Transformation Products from Polyvinyl Chloride Microplastics
into Aquatic Environments. Commun. Chem..

[50] Xu Z., Zhang J., Qi R., Liu Q., Cao H., Wen F., Liao Y., Shih K., Tang Y. (2025). Complex Release Dynamics
of Microplastic Additives: An Interplay of Additive Degradation and
Microplastic Aging. J. Hazard. Mater..

[51] Gulizia A. M., Patel K., Philippa B., Motti C. A., van Herwerden L., Vamvounis G. (2023). Understanding Plasticiser Leaching
from Polystyrene
Microplastics. Sci. Total Environ..

[52] Huang, J. ; Nkrumah, P. N. ; Li, Y. ; Appiah-Sefah, G. Chemical Behavior of Phthalates Under Abiotic Conditions in Landfills. In Reviews of Environmental Contamination and Toxicology; Whitacre, D. M. , Ed.; Springer: New York, NY, 2013, Vol. 224; pp 39–52. 10.1007/978-1-4614-5882-1_2. 23232918

[53] Prasad B. (2021). Phthalate
Pollution: Environmental Fate and Cumulative Human Exposure Index
Using the Multivariate Analysis Approach. Environ.
Sci. Process. Impacts.

[54] Liang D.-W., Zhang T., Fang H. H. P., He J. (2008). Phthalates Biodegradation
in the Environment. Appl. Microbiol. Biotechnol..

[55] Rummel C. D., Jahnke A., Gorokhova E., Kühnel D., Schmitt-Jansen M. (2017). Impacts of Biofilm Formation on the
Fate and Potential
Effects of Microplastic in the Aquatic Environment. Environ. Sci. Technol. Lett..

[56] Marttinen S. K., Kettunen R. H., Sormunen K. M., Rintala J. A. (2003). Removal
of Bis­(2-Ethylhexyl)
Phthalate at a Sewage Treatment Plant. Water
Res..

[57] Net S., Sempéré R., Delmont A., Paluselli A., Ouddane B. (2015). Occurrence, Fate, Behavior
and Ecotoxicological State
of Phthalates in Different Environmental Matrices. Environ. Sci. Technol..

[58] Guo H., Zheng X., Ru S., Luo X., Mai B. (2019). The Leaching
of Additive-Derived Flame Retardants (FRs) from Plastics in Avian
Digestive Fluids: The Significant Risk of Highly Lipophilic FRs. J. Environ. Sci..

[59] Yu Y.-X., Li J.-L., Zhang X.-Y., Yu Z.-Q., Van de
Wiele T., Han S.-Y., Wu M.-H., Sheng G.-Y., Fu J.-M. (2010). Assessment of the Bioaccessibility of Polybrominated Diphenyl Ethers
in Foods and the Correlations of the Bioaccessibility with Nutrient
Contents. J. Agric. Food Chem..

[60] López-Vázquez J., Rodil R., Trujillo-Rodríguez M. J., Quintana J. B., Cela R., Miró M. (2022). Mimicking Human Ingestion of Microplastics:
Oral Bioaccessibility Tests of Bisphenol A and Phthalate Esters under
Fed and Fasted States. Sci. Total Environ..

[61] Sixto A., El-Morabit B., Trujillo-Rodríguez M. J., Carrasco-Correa E. J., Miró M. (2021). An Automatic Flow-through System for Exploration of
the Human Bioaccessibility of Endocrine Disrupting Compounds from
Microplastics. Analyst.

[62] Deng Y., Yan Z., Shen R., Wang M., Huang Y., Ren H., Zhang Y., Lemos B. (2020). Microplastics Release Phthalate Esters
and Cause Aggravated Adverse Effects in the Mouse Gut. Environ. Int..

[63] Amara I., Timoumi R., Annabi E., Salem I. B., Abid-Essefi S. (2020). Di­(2-Ethylhexyl)
Phthalate Inhibits Glutathione Regeneration and Dehydrogenases of
the Pentose Phosphate Pathway on Human Colon Carcinoma Cells. Cell Stress Chaperones.

[64] Cousins I., Mackay D. (2000). Correlating the Physical–Chemical
Properties
of Phthalate Esters Using the “three Solubility” Approach. Chemosphere.

[65] Domínguez-Romero E., Scheringer M. (2019). A Review of
Phthalate Pharmacokinetics in Human and
Rat: What Factors Drive Phthalate Distribution and Partitioning?. Drug Metab. Rev..

[66] Chi Z., Zhao J., Li W., Araghi A., Tan S. (2017). In Vitro Assessment
of Phthalate Acid Esters-Trypsin Complex Formation. Chemosphere.

[67] Wang Y.-Q., Zhang H.-M. (2013). Comparative Studies
of the Binding of Six Phthalate
Plasticizers to Pepsin by Multispectroscopic Approach and Molecular
Modeling. J. Agric. Food Chem..

[68] Praveena S. M., Teh S. W., Rajendran R. K., Kannan N., Lin C.-C., Abdullah R., Kumar S. (2018). Recent Updates
on Phthalate Exposure
and Human Health: A Special Focus on Liver Toxicity and Stem Cell
Regeneration. Environ. Sci. Pollut. Res..

[69] Yan Z., Zhang S., Zhao Y., Yu W., Zhao Y., Zhang Y. (2022). Phthalates Released from Microplastics
Inhibit Microbial Metabolic
Activity and Induce Different Effects on Intestinal Luminal and Mucosal
Microbiota. Environ. Pollut..

[70] Hoang T. C., Felix-Kim M. (2020). Microplastic Consumption and Excretion by Fathead Minnows
(*Pimephales Promelas*): Influence of Particles Size
and Body Shape of Fish. Sci. Total Environ..

[71] Roch S., Ros A. F. H., Friedrich C., Brinker A. (2021). Microplastic Evacuation
in Fish Is Particle Size-Dependent. Freshw.
Biol..

[72] Roch S., Friedrich C., Brinker A. (2020). Uptake Routes of Microplastics in
Fishes: Practical and Theoretical Approaches to Test Existing Theories. Sci. Rep..

[73] Barboza L. G. A., Lopes C., Oliveira P., Bessa F., Otero V., Henriques B., Raimundo J., Caetano M., Vale C., Guilhermino L. (2020). Microplastics
in Wild Fish from North East Atlantic
Ocean and Its Potential for Causing Neurotoxic Effects, Lipid Oxidative
Damage, and Human Health Risks Associated with Ingestion Exposure. Sci. Total Environ..

[74] Elizalde-Velázquez G. A., Gómez-Oliván L. M. (2021). Microplastics
in Aquatic Environments:
A Review on Occurrence, Distribution, Toxic Effects, and Implications
for Human Health. Sci. Total Environ..

[75] Green D. S., Boots B., O’Connor N. E., Thompson R. (2017). Microplastics Affect
the Ecological Functioning of an Important Biogenic Habitat. Environ. Sci. Technol..

[76] Wootton N., Reis-Santos P., Gillanders B. M. (2021). Microplastic in Fish—A Global
Synthesis. Rev. Fish Biol. Fish..

[77] Hu X., Gu Y., Huang W., Yin D. (2016). Phthalate Monoesters as Markers of
Phthalate Contamination in Wild Marine Organisms. Environ. Pollut..

[78] McIlwraith H. K., Kim J., Helm P., Bhavsar S. P., Metzger J. S., Rochman C. M. (2021). Evidence
of Microplastic Translocation in Wild-Caught Fish and Implications
for Microplastic Accumulation Dynamics in Food Webs. Environ. Sci. Technol..

