# Supplementary material for: Leaching of Di(2-ethylhexyl) Phthalate from Polyvinyl Chloride Microplastics in Synthetic Freshwater, Seawater, and Fish In Vitro Digestion Fluids
Source: ACS ES T Water. 2026 Jul 21;6(8):5291–301. doi: 10.1021/acsestwater.6c00513 (PMC13488399; doi:10.1021/acsestwater.6c00513)
Supplement: Supplementary file 1 [file ew6c00513_si_001.pdf]

Supplementary Information: Leaching of di(2-ethylhexyl) phthalate from polyvinyl chloride microplastics in synthetic freshwater, seawater, and fish in vitro digestion fluids

Rui Cai[a], Parul Sharma[b], Victoria Rhodes[c], Dhanuka Thennakoon[b], Justin Scott[a], Jimmie Weaver[b], Matteo Minghetti[c], Jorge Gonzalez-Estrella[a*+]

[a]*School of Civil and Environmental Engineering, Oklahoma State University, Stillwater, Oklahoma, 74078, United States of America*

[b]*Department of Chemistry, Oklahoma State University, Stillwater, Oklahoma, 74078, United States of America*

[c]*Department of Integrative Biology, Oklahoma State University, Stillwater, Oklahoma, 74078, United States of America*

**Corresponding Author: Jorge Gonzalez Estrella*

Contents

1. Fish food ingredients....3
2. Identification of plasticizers in MPs in GCMS....4
   2.1 Total Ion Chromatogram (TIC) of Non-Aged PVC MPs....4
   2.2 Main peaks mass spectrum and the hint compounds....4
3. DEHA, DEHP, and DOTP: Physicochemical Properties, Quantifier/Qualifier Ions, and Analytical Limits....9
4. Leached additives relative to PVC mass....12
5. EDS spectrum of PVC MPs....13
6. Mass spectra of likely degradation products of plasticizers....14

1. Fish food ingredients

The fish food used in this study was Fluker's Medley Treat (Fluker Farms, LA), composed of freeze-dried river shrimp, mealworms, and crickets. According to the manufacturer's specifications, its proximate composition is as follows:

- Crude protein: ≥58%
- Crude fat: ≥12%
- Crude fiber: ≤7%
- Moisture: ≥8.75%

2. Identification of plasticizers in MPs

2.1 Total Ion Chromatogram (TIC) of Non-Aged PVC MPs

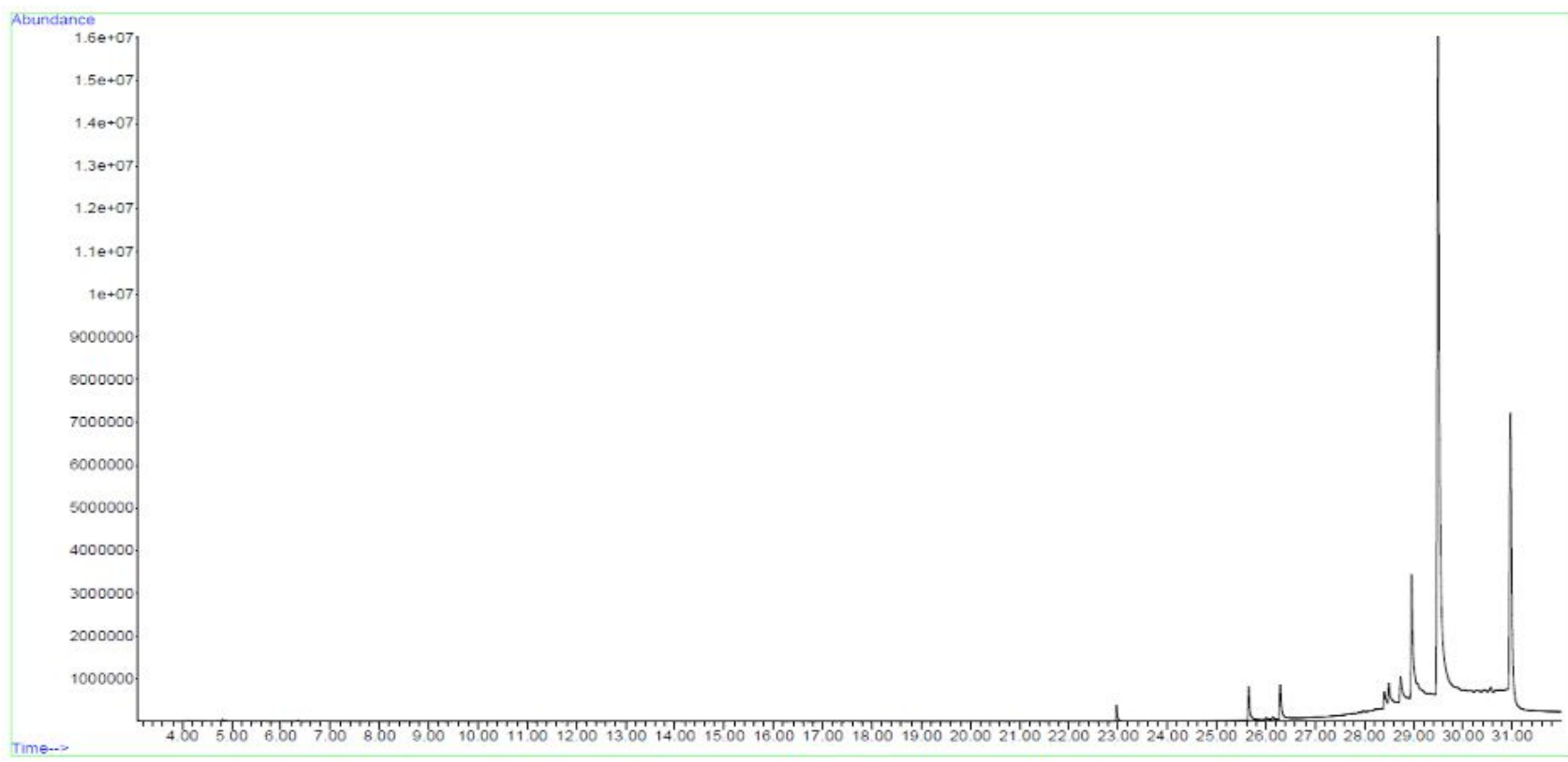

Fig. 1S. Total Ion Chromatogram (TIC) of Non-Aged PVC MPs (Full Scan Mode).

2.2 Main peaks mass spectrum and the hint compounds

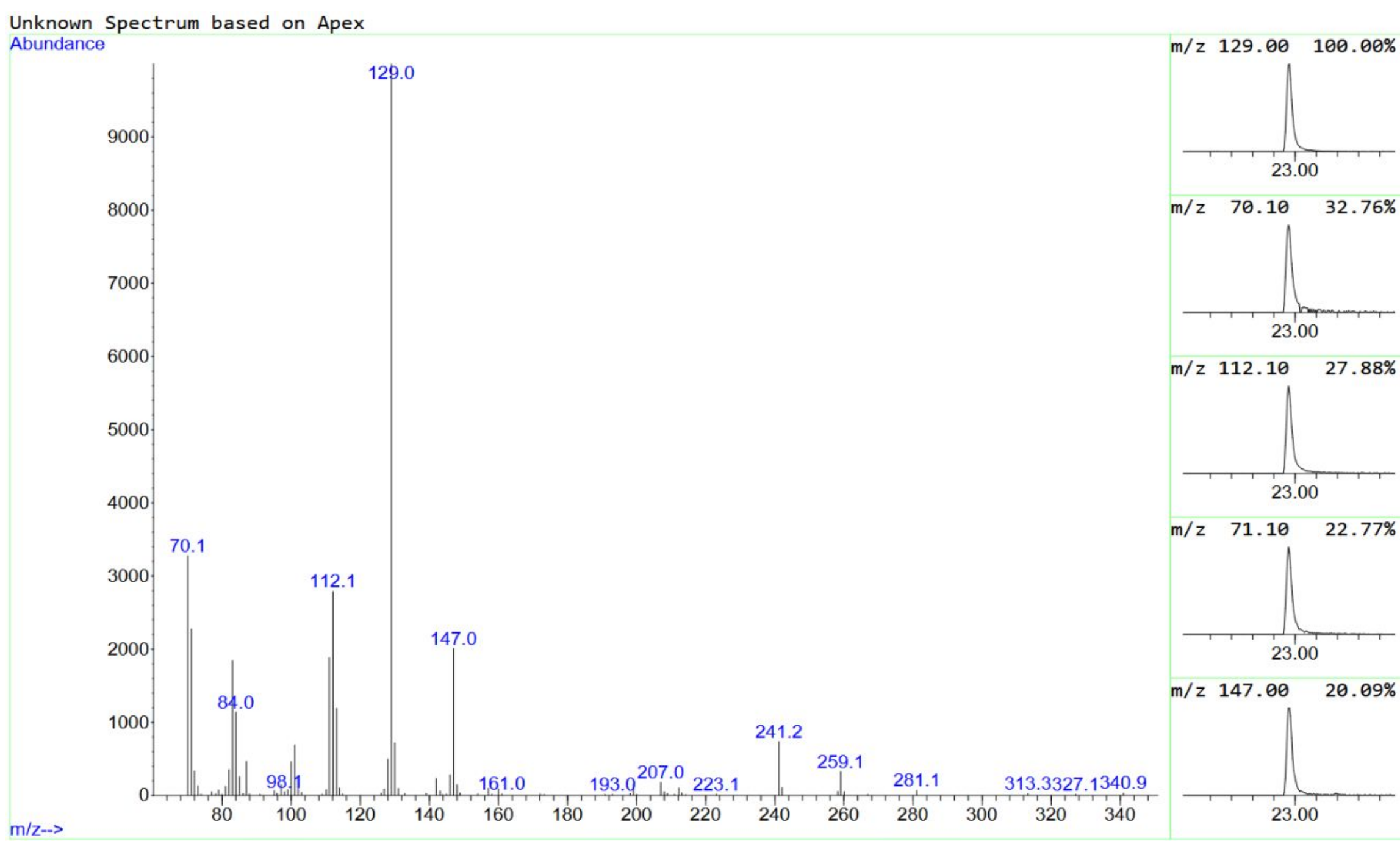

Fig. 2S. Mass spectrum of the peak at 22.9 min.

- Hit: Hexanedioic acid, bis(2-ethylhexyl) ester

Formula: $C_{22}H_{42}O_4$

MW: 370 Exact Mass: 370.30831 CAS#: 103-23-1

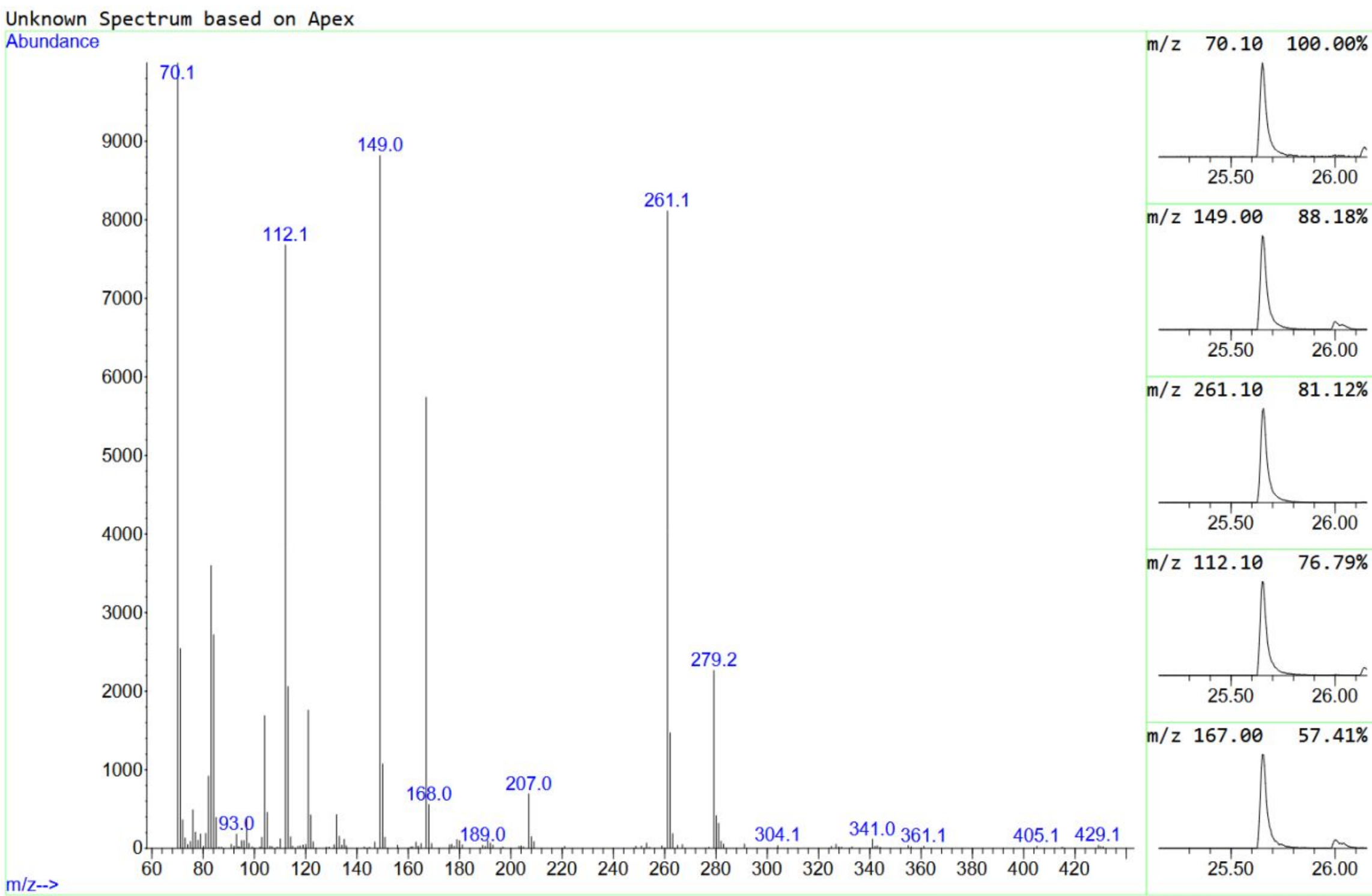

Fig. 3S. Mass spectrum of the peak at 25.6 min.

- Hit: 1,4-Benzenedicarboxylic acid, bis(2-ethylhexyl) ester

  Formula: $C_{24}H_{38}O_4$

  MW: 390 Exact Mass: 390.27701 CAS#: 6422-86-2

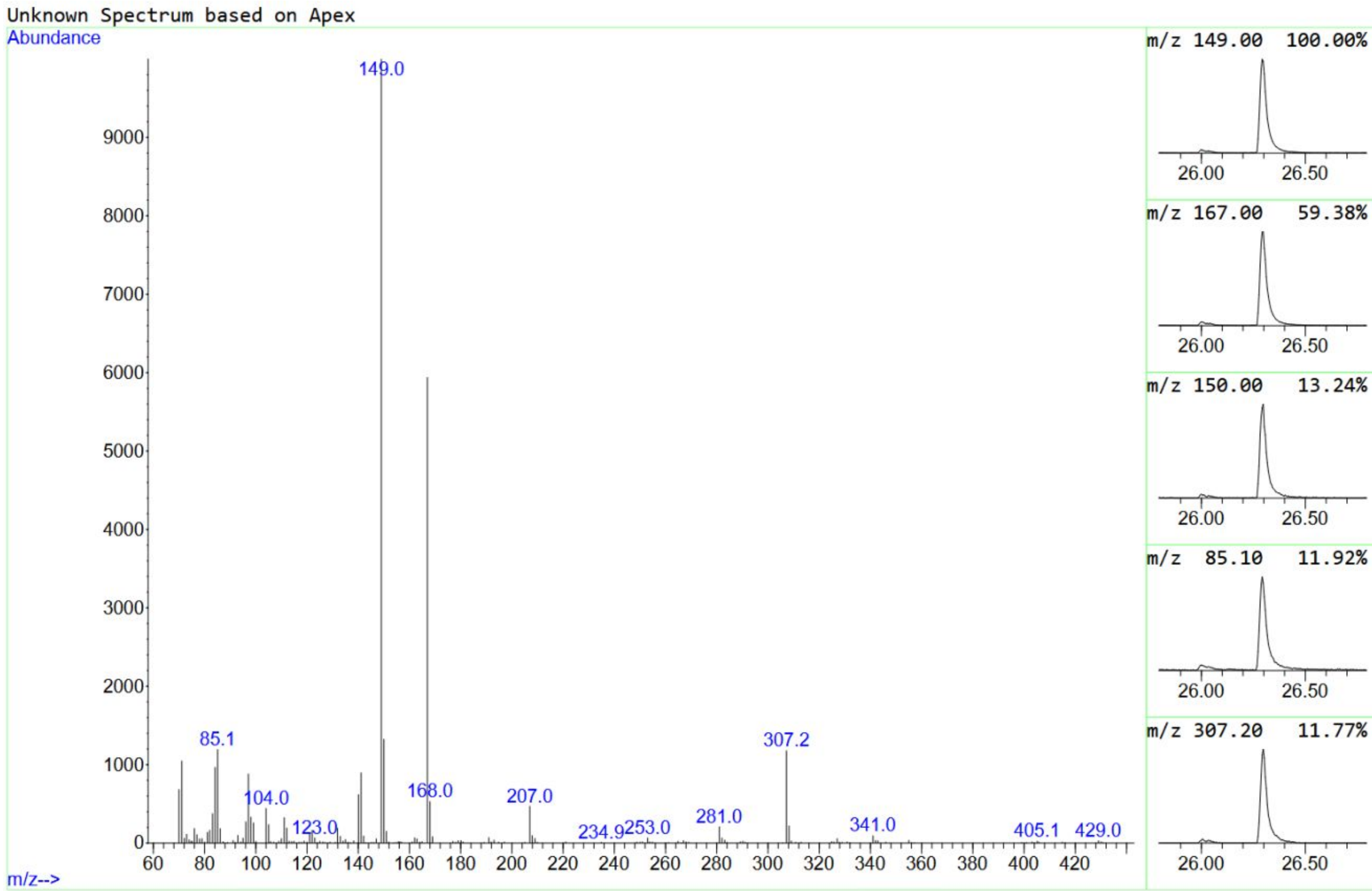

Fig. 4S. Mass spectrum of the peak at 26.3 min.

- Hit: Bis(2-ethylhexyl) phthalate

  Formula: $C_{24}H_{38}O_4$

  MW: 390 Exact Mass: 390.27701 CAS#: 117-81-7

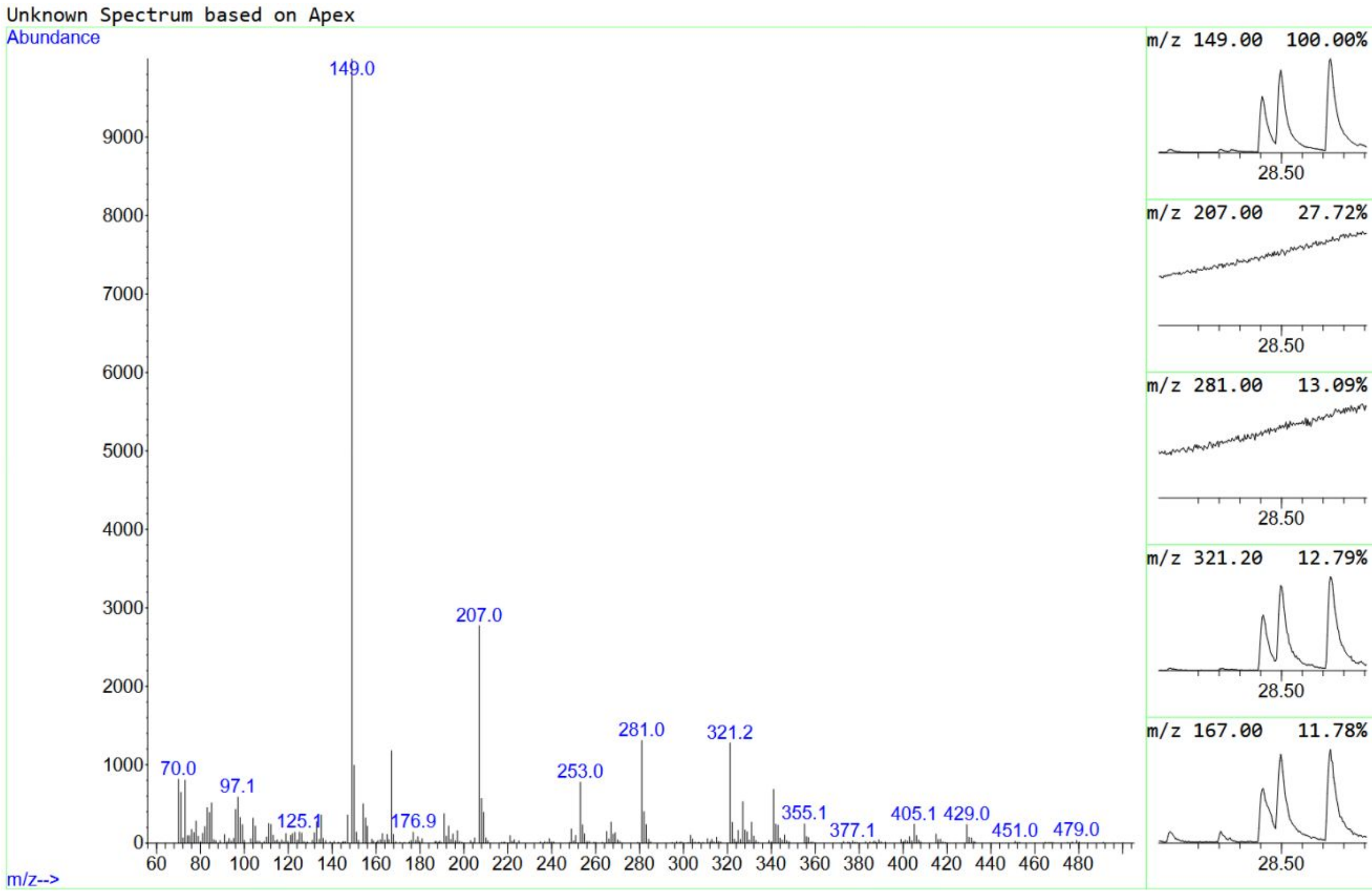

Fig. 5. Mass spectrum of the peaks at 28.3~30.0 min.

- Hit: 1,2-Benzenedicarboxylic acid, diundecyl ester

  Formula: $C_{30}H_{50}O_4$

  MW: 474 Exact Mass: 474.37091 CAS#: 3648-20-2

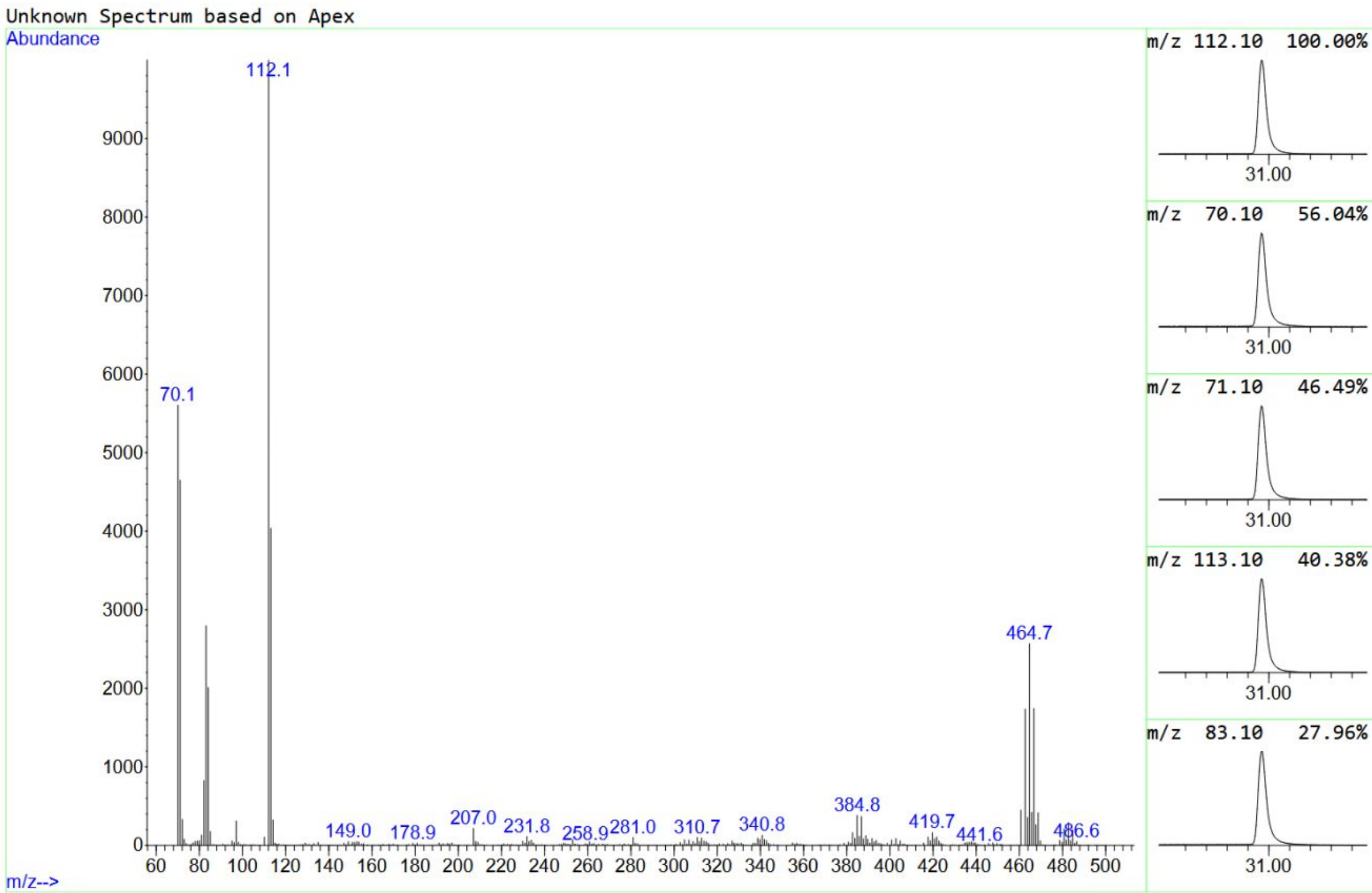

Fig. 6S. Mass spectrum of the peak at 30.9 min.

- Hit: Phthalic acid, tetrabromo-, bis(2-ethylhexyl) ester

Formula: $C_{24}H_{34}Br_4O_4$

MW: 702 Exact Mass: 701.91906 CAS#: 26040-51-7

3. Quality Assurance (QA) and Quality Control (QC)

Table S1. Physicochemical Properties, Selected Ions, and Detection Limits of DEHA, DEHP, and DOTP.

	DEHA	DEHP	DOTP
Solubility in water	0.78 mg/l at 22 °C	0.27 mg/L at 25 °C	4.0 mg/L at 20 °C
Log K_{ow}	6.1	7.6	8.39
Retention time (min)	14.5	16.1[1]	18.9[1]
Quantifier ion	129	149, 167[1]	149, 261[1]
Qualifier ion	147	167, 279	167, 261
LOD (µg/l)	4.16	8.21	8.98
LOQ (µg/l)	12.6	24.49	27.21

1: Since DEHP and DOTP are isomers, co-elution sometimes occurs during measurement. When this happened, m/z 167 was used as the quantifier ion for DEHP and m/z 261 for DOTP.

Table S2. Calibration and analytical parameters for DEHP quantification.

Analyte	DEHP
Instrument calibration range in final ACN extract (μg L^{-1})	100–1000
Equivalent original-sample range after 10× SPE concentration (μg L^{-1})	10–100
Number of calibration points	6
R^2	0.993
Retention time (min)	16.11
Quantifier ion	149 / 167*
Qualifier ion(s)	167, 279
Sample-equivalent LOD (μg L^{-1})	8.21
Sample-equivalent LOQ (μg L^{-1})	24.49

*The GC-MS calibration standards were prepared in ACN and represent concentrations in the final SPE extract. Because samples were concentrated by SPE at a 10:1 sample-to-eluent ratio, the corresponding original-sample concentration range was 10–100 μg L^{-1}. LOD and LOQ are reported as sample-equivalent concentrations after back-calculation to the original liquid sample. m/z 149 was used as the primary quantifier ion for DEHP; when interference or partial co-elution with DOTP occurred, m/z 167 was used for DEHP quantification.

Table S3. SPE recovery of DEHP.

Analyte	Test matrix	Spike level in original test solution (μg L^{-1})	n	Recovery (%)
DEHP	Synthetic freshwater	60	3	106.6 ± 4.3
DEHP	Synthetic seawater	60	3	104.9 ± 5.7
DEHP	Modified L-15 base buffer	60	3	96.3 ± 6.0

Note: The modified L-15 base buffer was used as the base medium for the fish *in vitro* digestion system. The recovery test in this buffer evaluates SPE recovery in the processed liquid phase but does not represent full recovery from the complete gastric or intestinal digestion matrices, which contain additional components such as pepsin, bile, pancreatin, and food particles. Spike samples were concentrated by SPE at a 10:1 sample-to-eluent ratio.

4. Leached additives recovered in the hexane extract relative to PVC mass

The mass percentage of each additive (%, w/w, relative to PVC MPs) was calculated as:

$$Mass\ percentage\ (\%) = \frac{C_{additive} \times V_{hexane}}{m_{PVC}} \times 100\%$$

where $C_{additive}$ is the concentration of the additive in the hexane extract (mg L^{-1}), V_{hexane} is the volume of the extract (L), and m_{PVC} is the mass of PVC microplastics (mg).

5. EDS spectrum of PVC MPs

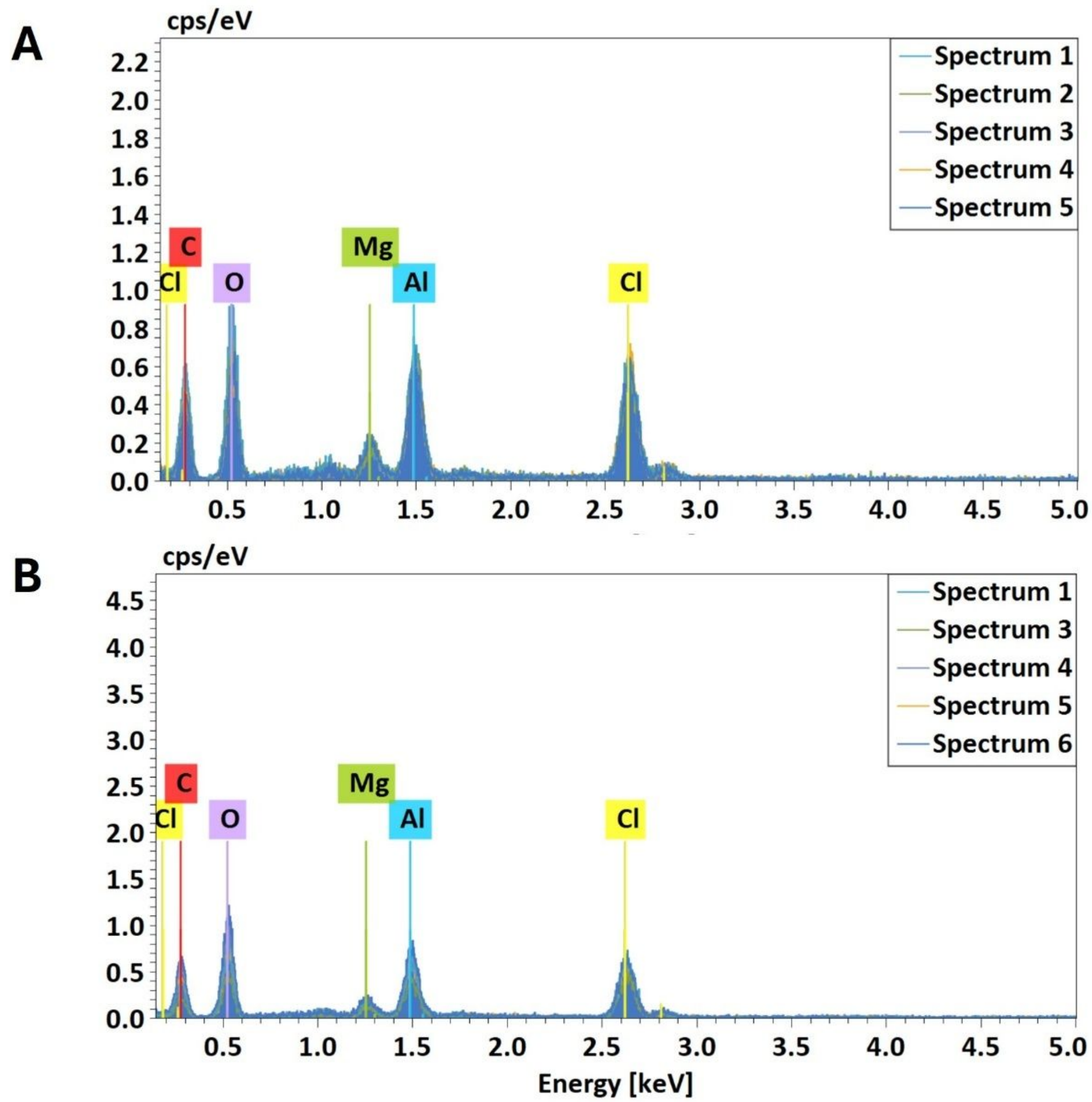

Fig. 7S. EDS spectrum of PVC MPs: (A) Non-aged PVC MPs, (B) UV-aged PVC MPs.

6. Mass spectra of likely degradation products of plasticizers

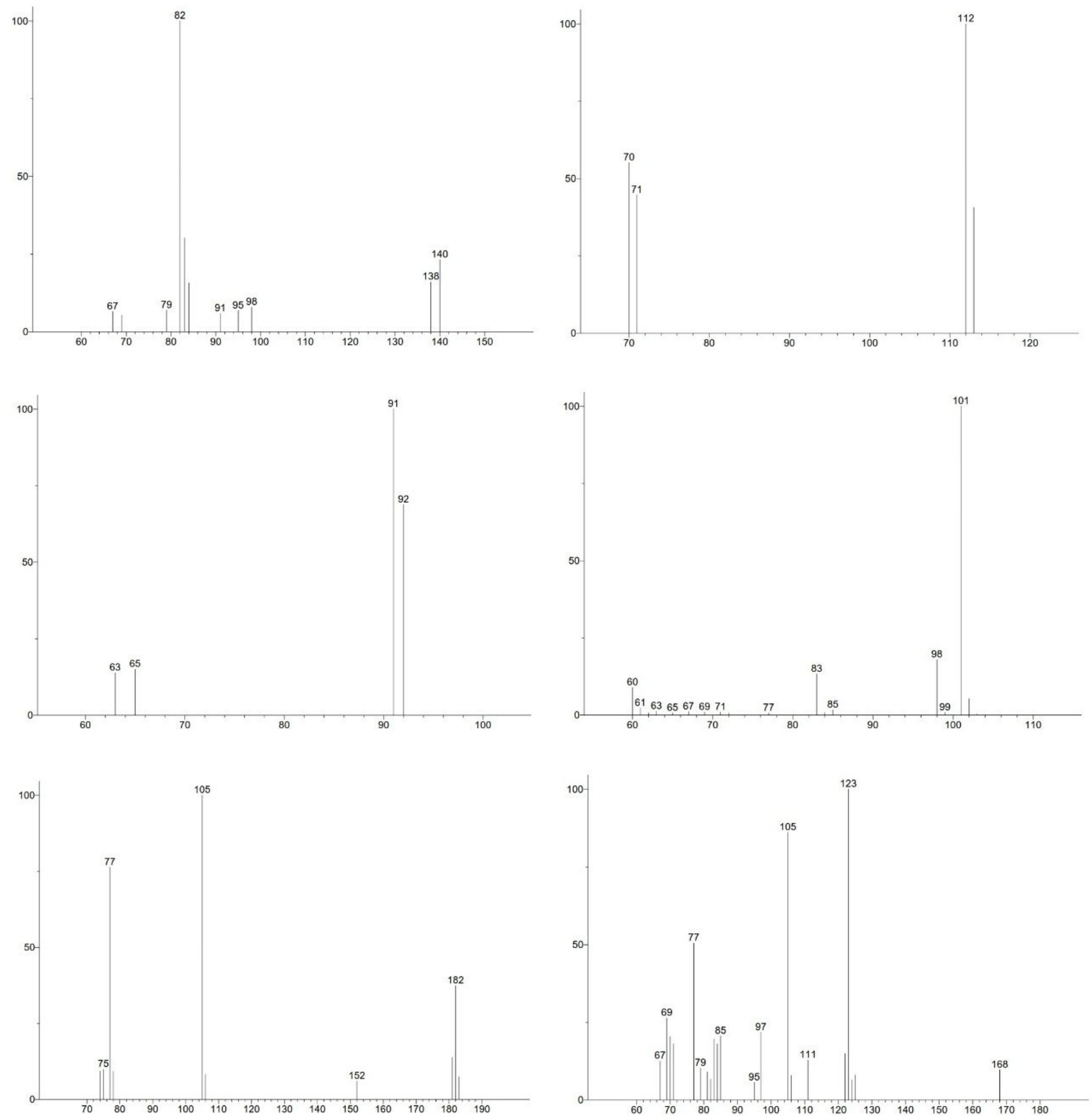

Fig. 8. Mass spectra of likely degradation products of plasticizers.
